# Health system preparedness for integration of mental health services in rural Liberia

**DOI:** 10.1186/s12913-017-2447-1

**Published:** 2017-07-27

**Authors:** Wilfred S. Gwaikolo, Brandon A. Kohrt, Janice L. Cooper

**Affiliations:** 1 The Carter Center Mental Health Program, Liberia Mental Health Initiative, Monrovia, Liberia; 2 Duke Global Health Institute, Duke University, Durham, USA

**Keywords:** Africa, Barriers to care, Developing countries, Global health, Health systems, Mental health, Primary care, Stigma

## Abstract

**Background:**

There are increasing efforts and attention focused on the delivery of mental health services in primary care in low resource settings (e.g., mental health Gap Action Programme, *mhGAP*). However, less attention is devoted to systematic approaches that identify and address barriers to the development and uptake of mental health services within primary care in low-resource settings. Our objective was to prepare for optimal uptake by identifying barriers in rural Liberia. The country’s need for mental health services is compounded by a 14-year history of political violence and the largest Ebola virus disease outbreak in history. Both events have immediate and lasting mental health effects.

**Methods:**

A mixed-methods approach was employed, consisting of qualitative interviews with 22 key informants and six focus group discussions. Additional qualitative data as well as quantitative data were collected through semi-structured assessments of 19 rural primary care health facilities. Data were collected from March 2013 to March 2014.

**Results:**

Potential barriers to development and uptake of mental health services included lack of mental health knowledge among primary health care staff; high workload for primary health care workers precluding addition of mental health responsibilities; lack of mental health drugs; poor physical infrastructure of health facilities including lack of space for confidential consultation; poor communication support including lack of electricity and mobile phone networks that prevent referrals and phone consultation with supervisors; absence of transportation for patients to facilitate referrals; negative attitudes and stigma towards people with severe mental disorders and their family members; and stigma against mental health workers.

**Conclusions:**

To develop and facilitate effective primary care mental health services in a post-conflict, low resource setting will require (1) addressing the knowledge and clinical skills gap in the primary care workforce; (2) improving physical infrastructure of health facilities at care delivery points; and (3) implementing concurrent interventions designed to improve attitudes towards people with mental illness, their family members and mental health care providers.

## Background

Access to mental health services is severely limited in Liberia, and yet in high demand [1, 2], Liberia’s health system, which included nominal mental health services limited to one psychiatric rehabilitation facility, was virtually destroyed during the 14 year civil war that ended in 2003. The conflict killed an estimated 250,000 people, resulted in massive population displacement, and left much of the population physically and emotionally traumatized [3]. Despite the post-war emergency situation, psychosocial and mental health supports were grossly inadequate, and the health infrastructure was devastated [4]. Of Liberia’s 550 pre-war health facilities, only 354 facilities (12 public hospitals, 32 public health centers, 189 public clinics, 10 private health centers and 111 private clinics) functioned by the end of 2003. Eighty percent of these were managed by non-governmental organizations (NGOs) and faith-based organizations (FBOs) [4]. Additionally, many in the health workforce, including doctors, nurses, and other health workers, fled the country during the war. A rapid assessment of the clinical workforce including private, NGO, and government workers estimated that the total workforce was 3107 persons: 168 physicians, 273 physician assistants (PAs), 443 registered nurses (RNs), and more than 1000 nurse aides; this workforce was mismatched and clustered around the capital Monrovia [4]. More than a decade after the civil war, the health system remains fragile and still faces enormous challenges.

The geographical disparity in health care availability extends to mental health service provision. A 2008 survey based on self-report found that 40% of the population has symptoms that are consistent with major depression and 44% has symptoms consistent with post-traumatic stress disorder [3]. However, other studies have found that less than one-third of health care facilities provide mental health care. According to a 2011 government accreditation survey, only 18% of health care facilities in Liberia reported having a health care worker trained to provide mental health services, with those few concentrated in urban areas [5, 6].

To address the gap between the demand for services and the paucity in supply, often termed “the treatment gap”, the Liberia National Mental Health Policy calls for a decentralized approach to mental health care, and improvement in the quality of lives of all patients and families. An explicit goal of the policy and its strategic plan is to increase access to health services. The infrastructure component of the National Health Plan emphasized geographical accessibility through a decentralized system of health clinics, health centers, and hospitals [7]. This was informed by the fact that roughly 40% of the population in Liberia live more than 5 km away from the nearest health facility.

One of the solutions to the gap in mental health services has been to train cadres of specialist mental health nurses and physicians assistants through a six-month training course including didactic and clinical components. This training program has been conducted twice yearly since 2011 by the Government of Liberia Ministry of Health in collaboration with The Carter Center Mental Program-Liberia Initiative. As of 2015, there were 144 trained nurses and physician assistants, who are licensed by the government to practice independently to provide mental health services in the primary care setting. These professionals are known as mental health clinicians. In addition, two other international non-governmental organizations have also trained approximately 20 mid-level providers in mental health. Collectively, these workers constitute the ‘specialist cadre’ to meet the needs of people with severe mental disorders and epilepsy (PWSMDE).

Although developing a cadre of providers with intensive 6-month training in mental health has been a step toward reducing the treatment gap, this has been insufficient to assure access to mental health care in all primary care settings throughout the country. One approach proposed by the World Health Organization (WHO) to address this need has been implementation of the mental health Gap Action Programme (mhGAP), which is an initiative for brief (i.e., 1 week) training for primary care workers [8]. Prior studies have demonstrated the effectiveness in low resource settings of programs to integrate mental health into primary care [9, 10] and to deliver psychological treatments by non-specialists [11].

Similar formative assessments in other post-conflict low-income countries has identified similar challenges related to stigma [12]. The barriers related to stigma are not limited to post-conflict settings, but have been identified throughout low- and middle income countries (LMICs) [13].

Though there are increasing efforts worldwide for the delivery of mental health services in primary care in low resource settings with guidelines such as mhGAP, there remains less attention to systematic approaches to identify and address barriers to development and uptake of primary care-based services, especially in low-resource, non-Western cultural settings. Lack of funding for providers to deliver mental health services, lack of access to medications, stigma against persons with mental health problems, and lack of political will have all been identified as potential barriers to implementation of primary care mental health services [14–16]. Similar barriers have been identified in Liberia; for example, a qualitative assessment in four Liberian communities found that one-third of respondents did not believe that an individual with mental illness could engage in any income-earning activity [17]. In the same study, between 75 and 100% of respondents endorsed the notion that people with mental illness affected their family and/or community’s economy negatively. Lack of transportation, training, and coordination across health, social, and security sectors are also barriers in Liberia [18].

Therefore, our goal in this study was to employ mixed methods to identify potential barriers to development and uptake of mental health services for an mhGAP-based primary care program in rural Liberia.

## Methods

### Setting

The program Mental Health Beyond Facilities (mhBeF) is funded through a three-year grant awarded to Makerere University School of Public Health from Grand Challenges Canada. The objective of mhBeF is to develop and implement an evidence-based comprehensive community-based mental health services (CCMHS) package in accordance with the mental health Gap Action Programme (mhGAP) for persons with severe mental disorders and epilepsy (PWSMDE) in Liberia, Uganda, and Nepal. As post-conflict countries, Liberia, Uganda, and Nepal each have high burden of mental disorders and a lack of community-based mental health services. The mhBeF project is implemented in Liberia by The Carter Center Mental Health Program, in partnership with the Government of Liberia Ministry of Health and Social Welfare.

mhBeF was designed with two phases: (1) a formative phase of mixed methods research to assess the health system preparedness for integration of mental health care, and then adapt the CCMHS package according to the health system and cultural context; and (2) a pragmatic trial comparing regions where health facilities integrate mental health services versus regions where no additional mental health services were integrated. In the formative phase, each countries’ research team examined domains considered central to the successful development and implementation of CCMHS, including a proposed mobile health component. The purpose of the formative phase was to inform the local adaptation and implementation of the CCMHS package, and focused on lessons learned in the development of this package of care with potential for replication to other post-conflict and diverse cultural, economic, and low resource settings. After conclusion of the formative phase, the pragmatic trial would compare regions where health facilities would have mental health services integrated (implementation sites) and regions where health facilities would not receive additional services (control sites). The sites would be compared with regard to patient outcomes after approximately 12–18 months of services.

**Fig. 1:**
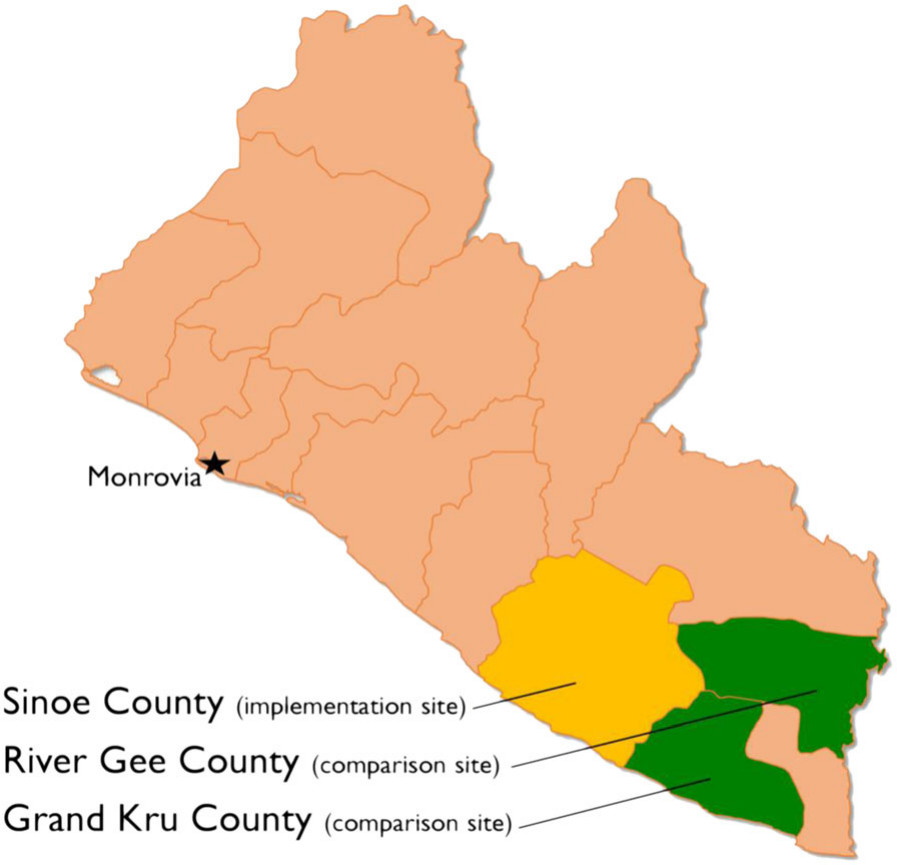
Map of Liberia and research sites

The formative research in Liberia was conducted in three of country’s 15 counties: Sinoe, River Gee and Grand Kru in southeastern Liberia (see Fig. 1). Sinoe was selected as the project implementation site by the Ministry of Health because it exemplified regions with low performance in government health facilities’ accreditation processes. If the mhBeF program could be adapted for and successful in Sinoe, then it was assumed that it could be scaled-up throughout the country including to other regions with low performing health facilities.

Sinoe county has approximately 111,267 inhabitants [19]. The major languages spoken are Liberian English, Kru, Sarpo, Krahn and Bassa. The county’s health infrastructures consist of one hospital, F. J. Grante Hospital, which is located in the county capital Greenville City, and 32 clinics. Of the 32 clinics, the government’s County Health Team supports 22 clinics while international non-governmental organizations support 10 clinics. Thirty-nine percent of the population lives within 15 km walking distance (3–4 h) to a health facility, suggesting limited access to facility-based health services. There are approximately 719 catchment localities for the 32 health facilities of which 10 clinics were selected for the mhBeF project, which covers 43% of the health facilities’ catchment area. Half of the mhBeF project catchment localities are situated outside of a five-kilometer buffer from their nearest health facility in order to evaluate the penetration of the services for remote populations. Sinoe county was the site of both health facility profile data collection and additional stakeholder qualitative interviews.

Two adjacent counties, River Gee and Grand Kru, were selected as control sites for the subsequent pragmatic trial. The control sites share similar geography, face similar health care conditions, and share similar demographic characteristics as Sinoe. As recorded by the 2008 National Population and Housing Census, Grand Kru has a population of approximately 58,000. River Gee has a population of 66,789. Four health facilities under the supervision of Mental Health Clinicians (MHCs) in Grand Kru and five facilities in River Gee were identified as controls. The control facilities participated in the health facility assessment, but no additional stakeholder qualitative interviews were conducted in the control counties.

### Health facility profiles

Health facility profiles were completed in the implementation county (Sinoe) and the two control counties (Grand Kru and River Gee). Health facility profiles were conducted in three counties (Sinoe, Grand Kru and River Gee) to obtain descriptive statistics of resources and services. To complete health facility profiles, three research assistants interviewed the administrator of the health facility, known as “Officer-in-Charge” (OICs). The health administrators provided information on the government categorization of the type of the health facility (e.g., hospital, hospital-affiliated health center, health clinic, dispensary, walk-in-surgery clinic), managing authority (e.g., government, private for-profit, private non-profit); catchment area and average distance of travel to the facility; facility infrastructure (building designed for health facility use only vs. conversion of non-health structure into health facility; condition of walls; condition of floor; working phone or short-wave radio; electricity; water; toilet), clinic consultation space (e.g., number of examination rooms; availability of separate rooms for confidential consultation); health management information systems (HMIS) data (e.g., number of patients with neuropsychiatric disorders provided with service in the past month); supervision system, health service personnel (e.g., number and type of staff, prior training in mental health); availability of mental health services; availability of psychiatric medications; and availability of protocols or guidelines for mental health treatment.

Health facility profiles were conducted in collaboration with the Government of Liberia county health teams. The county health teams nominated health facilities for participation in the health facility profile. Facilities in communities with more mental health needs and hard to reach facilities were nominated by the county health teams. Health facility administrators did not receive any financial compensation for completing the facility profiles.

### Stakeholder focus group discussions and key informant interviews

In the implementation county (Sinoe), qualitative data collection was included to supplement the health facility profiles. The qualitative data collection included focus group discussions (FGDs), and key informant interviews (KIIs) with health workers, mental health service users, health organizations^1^ and health facilities^2^ and other stakeholders. FGDs explored what and how services were utilized, as well as perceptions and factors that contributed to individuals’ access to and provision of mental health services and supports. KIIs were conducted with stakeholders and specialists. Purposive sampling was used to select participants with rich information on a particular topic to participate in KIIs and FGDs. We anticipated collecting six KIIs per group: health facility staff, health organizational leaders, service users, caregivers, and community members. A sample size of six per group was determined based on findings that basic metathemes emerge at this level [20]. Six FGDs were planned to compliment the KIIs, and this sample size was determined based on the intent to have two FGDs per stakeholder group: health facility staff, service users and caregivers, and community members. Two FGDs per stakeholder group would allow comparison of commonality [21]. FGDs and KIIs lasted approximately 90 min. All participants received a bar of soap and wristband in appreciation for their participation.

### Data collection

Four trained research assistants collected qualitative data through focus group discussions and key informant interviews as well as interviews with officers-in-charge of health facilities and observation of structural conditions of facilities.

Structured and semi-structured interview guides were developed based on the research objectives prior to data collection. In both FGDs and KIIs, research assistants read participants interview questions and presented vignettes; therefore, participants did not need to be literate. All focus group discussions and key informant interviews were conducted in Liberian English and recorded using a digital recording device. The interviews were then transcribed and merged with field notes. An independent transcriber also listened to audio recordings and reviewed and cleaned the data where appropriate to ensure accuracy in written transcripts.

### Data analysis

A framework analysis approach was used to analyze data. The researchers sought to answer specific questions that would inform the development of the CCMHS program. Through the framework analysis approach, we established patterns and structures in order to generate explanations of health system readiness and barriers to integration [21]. After transcription (Step 1 of framework analysis), we followed the subsequent framework analysis steps: 2-familiarization with the interviews, 3-coding, 4-developing a working analytic framework, 5-applying the analytical framework, 6-charting data into the matrix framework, 7-interpreting the data [22]. During familiarization with the interviews, we used an iterative reading to generate themes and explored the association among themes. Based on these, a coding framework was developed and tested on 25% of randomly selected transcripts and a final framework was developed for data coding. FGDs were analyzed for themes related to service availability and utilization, and mental health perceptions. Other factors that influenced access to care were also addressed. KIIs explored the following themes and issues: health care workforce, new cadre of workers, availability of psychotropic medicines and stigma. Data analysis was conducted by the three authors, with supplemental support from others who are named in the acknowledgement. The three authors coding the data include two native Liberians, one with a master’s degree in public health and one with a PhD in health services research, and an American with a PhD in anthropology and psychiatry board certification. The types of respondents represented expertise in the following areas: health organization, health facility, service users, family members, policy makers, and other key stakeholders. The analyses presented here address one theme and related codes from qualitative analyses: potential barriers to integration of mental health services in primary care.

Qualitative data were coded and analyzed using NVivo 10 software [23]. Quantitative data were managed using Microsoft Access and Excel applications and complete frequency of all variables was generated using STATA software.

## Results

Table 1 and Table 2 depict profiles of KII participants and of FGD participants respectively. Six FGDs and 22 KIIs were conducted during a formative research. Due to the diversity of occupational cadres at health facilities, we increased the number of health facility KIIs from six to eight. We conducted seven community stakeholder KIIs with individuals representing diverse perspectives on engaging with persons with mental illness. There was limited availability of health organization leaders, therefore we completed three KIIs with them. We conducted four KIIs with service users and family; additional KIIs were not seen as required because of common themes that were identified through two FGDs with service users and family members. Only one health facility KII was conducted due to resonance of themes with the KIIs. Three community stakeholder KIIs were conducted to capture the diversity of stakeholder perspectives. Facility profiles of 19 health facilities assessed available services, health workforce and facilities’ infrastructure. See Table 3 for summary of facilities.

**Table 1:** Key Informant Interview (KII) Participants (*n* = 22)

Recruitment Group	Occupation	Gender	KII reference #
Health Facility	Pharmacist	Male	1
Health Facility	Nurse Supervisor	Female	2
Health Facility	Nurse Supervisor	Female	3
Health Facility	Logistics Officer	Male	4
Health Facility	Health Administrator	Male	6
Health Facility	Officer in Charge/Health Care Worker/Midwife	Female	9
Health Facility	Mental Health Clinician	Female	17
Health Facility	Health Care Worker/Nurse	Male	18
Health Organization	Dispenser/Nurse	Male	5
Health Organization	Nurse/District Health Officer	Male	10
Health Organization	Service Head/ Psychosocial Officer	Male	11
Service User	Service User	Female	13
Service User	Service User	Female	14
Service User	Service User	Male	22
Family Member	Family Member	Female	15
Community Stakeholder	Traditional Leader	Male	7
Community Stakeholder	Religious Leader	Male	8
Community Stakeholder	Police Officer	Male	12
Community Stakeholder	Religious Leader	Male	16
Community Stakeholder	Policy Maker	Male	19
Community Stakeholder	Leader, Disability Union	Male	20
Community Stakeholder	Community Health Volunteer	Male	21

Note: Health facility refers to frontline primary health care workers, e.g., mental health workers, nurses, physician assistants, midwifes, pharmacists and support workers. Health organization refers to district/county level policy makers, planners, service heads, coordinators or administrators who provide support through supervision

**Table 2:** Profile of Focus Group Discussions (FGD)

FGD#	Category of Participants	Number of Participants	Average Age
1	Community	11	48 years
2	Service Users & Family Members	7	41 years
3	Community	8	36 years
4	Health Facility	7	35 years
5	Community	7	48 years
6	Service Users & Family Members	9	33 years

**Table 3:** Facility survey

Service and infrastructure	Sinoe County (*n* = 10)	Grand Kru County (*n* = 4)	River Gee County (*n* = 5)	Total (*n* = 19)
Access to ambulance	5 (50%)	2 (50%)	3 (60%)	53%
Access to phone	1 (10%)	1 (25%)	1 (20%)	16%
Assigned mental health care provider in facility	1 (10%)	2 (50%)	2 (40%)	26%
Electricity	4 (40%)	2 (50%)	4 (80%)	53%
Facilities with fence	1 (10%)	2 (50%)	1 (20%)	21%
Facilities with psychotropic medication	1 (10%)	2 (50%)	2 (40%)	26%
In-patient services	1 (10%)	2 (50%)	2 (40%)	26%

Analyses conducted around the major lines of inquiry showed that recurring themes clustered around the following areas: 1) the current mental health situation in Sinoe County and southeastern Liberia; 2) knowledge and attitudes of mental health; 3) the healthcare workforce; 4) pathways to care; 5) access to psychotropic medication; 6) stigma; 7) structural conditions of health facilities; and, 8) distances covered to access care.

### Perceptions of mental illness

Participants in key informant interviews and focus group discussions responded to questions about the “mental health situation” within communities. When asked about the different kinds of mental illnesses, epilepsy and depression were most frequently identified. Participants also pointed to drug abuse, schizophrenia, post-partum psychosis, and anxiety as types of mental illness commonly seen within the communities. The quote below flags the extent to which epilepsy and other severe mental health problem are of pressing concerns.

“*Yea for epilepsy, I think about 75% of the cases I received in let’s say October and up to now are epilepsy.*” ***(KII 17 – Mental Health Clinician)***

Over half of the key informants and several members of the focus group discussions identified or endorsed “frustration”^3^ and “African Science”^4^ as the major causes of mental illness. A majority of participants in the service users’ focus group discussions expressed that malaria was the cause of epilepsy. Witchcraft, demonic possession, and the “just world” retribution were also cited as causes of mental illness.

“*That’s what I am saying, some say African Science can cause the epilepsy; it can cause the brain [problem] even the mental problem we are talking about, this is Africa.*” ***(FGD 06 – Service Users/Family members)***

“*Sometimes when you do something bad to someone, they [are] able to go to someone then you get mental problem.*” ***(KII 22 – Service Users)***

“*Some people say malaria can do it; they say malaria can cause that same thing called epilepsy.*” ***(FGD 02– Service Users)***

### Access to and use of mental health services

Participants were asked about the availability and use of formal mental health services and the referral system within Sinoe County and the southeast. Overall, nearly all participants were familiar with the referral pathways for health care, but not necessarily for care for PWSMDE. They cited a wide range of referral sources, including primary health care facilities mainly around Greenville district in Sinoe County. They also named the county hospital (F.J. Grante Hospital), traditional healers, and religious leaders as sources of referral. In the case of crisis or serious mental illness, participants stated that individuals would be referred to Monrovia, the capital and approximately 340 km and 7 h drive away and/or the Jackson F. Doe Memorial Hospital in Tappita, Nimba County, approximately 180 miles and five-hour drive. Participants cited lack of transportation, a systemic lack of capacity, lack of drugs and costs of services as potential barriers to care. This was reinforced by health facility survey in which only 26% of 19 health facilities assessed in three counties reported having qualified mental health workers to provide service. Of 10 facilities in Sinoe, only 10% had a qualified mental health worker. According to respondents, although facilities with mental health workers were willing to provide mental health services, access to psychotropic medications were stated to be a serious challenge.

### Structural conditions and distance to facilities

Most people walked to facilities (84%) and only 53% of facilities reported easy access to a functional ambulance. The average distance to the nearest health facility in the study is 6.8 km. Some facilities have catchment localities that are more than 25 km away. Population distribution across localities and across facilities is uneven. The southern region of Sinoe is by far the most congested and probably the most densely populated region in the county, mhBeF project facilities serve at least 33% of 115,753 inhabitants in Sinoe. Half of all health facilities in the study have limited or no access to cellular phone network. Moreover, only 17% percent of all health facilities reported access to a phone.

The facility profile assessed the structural conditions of facilities. Of the 19 health facilities assessed in both the control and intervention arms, only 17% were enclosed by fence while 11% were in urgent need of repair on the floors. Seventy-two percent had functioning latrine while 11% reported access to continuous power supply. In the control sites, the percentage was much higher (22%) while in the intervention arm, none of the clinics reported access to continuous power supply. Fifty-three percent of facilities reported access to electricity, however; of these, only 11% had continuous supply of electricity. None of the facilities reported a designated room for confidential consultation.

### Mental health workforce

Participants discussed the health care workforce’s knowledge of mental health (inclusive of training curricula and needs) and the willingness of trained health care workers to provide mental health care. The majority of participants indicated that health care workers are willing to provide mental health services to individuals in need. However, many barriers to service provision were cited. These included little or unaccredited training, low incentives (no motivational packages, low salaries, and a high workload), and most importantly, a lack of drugs. Respondents stressed that the drug shortage was due to a weak supply chain, and deplorable road conditions between Monrovia and Greenville. Analysis of health facility baseline data also show that in Sinoe, the proposed intervention site, only one out of the ten facilities assessed reported having a trained mental health worker. In the other counties, two of the four facilities assessed in Grand Kru and two of five facilities in River Gee reported having trained mental health workers assigned.

“*I don’t think somebody will complain because there are other things that we are doing. We are doing HIV programs, we [are] doing TB and leprosy program, why can’t [we] integrate mental health into the PHC [primary health center] and take it as a full time* *responsibility? I don’t see it as a problem, the nurses will do that.*” ***(KII 09 – Health Facility/Midwife)***

“*We may not be mental health clinicians but at least we got some basic ideas from school. We did theory for 12 hours a month for three months, so we know that when someone is having a disorder.*” ***(KII 02 – Health Facility/Nurse Supervisor)***

“*I did psychiatry as a course. Well, I have not had any formal training in mental health, it helped me in that, at least I can be able to identify some mental health cases and be able to refer patients to the responsible people that were trained to carry on.*” ***(KII 18 – Health Facility/Nurse)***

### Availability of psychotropic medications

Availability of psychotropic medication at health facilities was also assessed. When asked about pharmaceutical needs and the availability of psychotropic medications, participants reported that medicines were scarce. Some health workers reported that phenobarbitone was used for epilepsy and that diazepam, amitriptyline, and carbamazepine were used to treat PWSMDE. All participants reported that medicines were to be provided free of charge at all primary care facilities. However, they reported that because of the weak supply chain, and lack of transportation, medication was not being readily transported from the capital Monrovia to primary care facilities. The facility baseline also revealed frequent stock-outs of mental health drugs and commodities at all facilities assessed. Only one facility in Sinoe with an assigned Mental Health Clinician reported having access to mental health medication, but reported frequent stock-outs. Of the nine facilities in the River Gee and Grand Kru, only four reported access to mental health medication.

*“The lack of appropriate drugs to be administering, and there are lot of needed resources like logistics, like the drugs will be available in Montserrado but to get it to this county is a problem, logistics is one of the key things.*” ***(KII 11 – Health Office/Nurse)***

### Stigma

Negative attitudes towards PWSMDE were identified throughout the FGDs and KIIs. The majority of study participants referred to PWSMDE using local stigmatizing idioms and insults such as “crazy”, “mad people”, “zepsi”, “cracky”, and “sarkar”. In addition to being called stigmatizing names, participants confirmed that PWSMDE are mistreated in their families and communities. The examples provided ranged from being mocked and teased to being shunned, denied jobs, chained, forced to work for little or no pay, and being beaten. Participants also reported that health care workers stigmatized PWSMDE. One participant reported that mental health workers are also stigmatized, and referred to as the “Crazy Doctors”. The participant reported, “*Because we deal with people with mental illness, so we are stigmatized*” (KII 17 – Health Facility/Mental Health Worker).

“*When you reach in some villages, their relatives …. because they have mental problem they are tied, or chained, so because they don't know where to go and where they can get some help or assistance rendered for that person, they will just keep that person into the village on chain, tied up.*” ***(KII 18 – Health care worker)*** *.*

“*For the others, like you are health worker but you don't have the [mental health] training, they [are] always pushing them [PWSMDE] away, they don't want to even be acquainted with them, that negative impact, that negative feeling, it’s in their mind, because they don't have formal training.*” ***(KII – 17 – Health Facility/Mental Health Worker).***

## Discussion

This study explored contextual factors and potential barriers to the development of primary care based mental health services in a low-resource, post-conflict, non-Western setting. It was designed to inform the development of a comprehensive community-based mental health services package. The main findings were; (i) poor or no mental health resources, lack of mental health knowledge in the community, in families, and among individuals; (ii) inadequate number of facilities close to the population; and, (iii) stigma. The poor or limited mental health resources were characterized by lack of trained mental health workers and lack of drugs, while the inferior health infrastructure capacity included facilities in disrepair, without access to basic sanitation and limited electricity and water supply. In addition, patients are compelled to travel long distances to health facilities. Poor access to qualified and skilled mental health providers led to participants overstating the range of available mental health resources. Some of the mental health referrals that respondents identified did not provide mental health services although they may have been good resources for general health. For example, participants cited Jackson F. Doe Hospital in Tappita as a referral hospital for mental health. While the hospital is a state-of- the art tertiary health facility in rural Liberia, it does not provide mental health services.

Providing accurate, simple information on the referral pathway for access to mental health services and emergencies would encourage and facilitate service use. In addition, it may reduce the likelihood that individuals and families would give up on seeking care, especially emergency care. Another key finding is the negative attitudes about mental health and stigma towards PWSMDE, their family and mental health workers. These findings are crucial to understanding what barriers exist and which services and supports are needed for uptake in Sinoe. These barriers, while not unique to mental health service delivery, included, poor infrastructure, lack of access to basic sanitation, poor referral pathways and knowledge of referral options and long distances to access care. Our findings suggest that mental health education and psycho-education must include information about the referral pathway for mental health services and the types of workers PWSMDE will access at every level of care.

This study also documents that general knowledge and belief about mental health among participants did not always conform to a bio-medical approach thereby raising the need for a more intensive and culturally sensitive mental health awareness. Some participants’ reported that they believed that witchcraft, demonic possessions and “just world” retribution cause mental illness. Some participants reported that maltreatment of the mentally ill and individuals with epilepsy remain pervasive in certain areas. Participants also indicated that choice of care depended upon what people felt was the cause of mental illness; signifying that they were more likely to seek traditional or spiritual care than medical care if they believed that the cause of illness was spiritual. Consequently, development of mental health services must attend to the educational and cultural factors associated with mental health conditions in low-income communities. This suggest that take-up of mental health services could benefit from intensive and culturally appropriate mental health education at the community and facility level.

This research confirms that many primary health care workers lack sufficient training in mental health. Although some health care workers reported completing course work in psychiatry during their training, this knowledge does not appear sufficient to support independent screening, diagnosis and treatment for PWSMDE. The baseline facility survey also showed that Sinoe County, with over 100,000 inhabitants at the time of this research, had only four trained Mental Health Clinicians to care for their mental health needs. As documented in other studies, this scarcity of mental health specialists affirms the overall shortage in specialist mental health personnel in LMICs and underscores the need for task-sharing mental health services [14]. Of note, in a study of mental health care seeking in Haiti, the lack of competency of health workers was found to be a bigger barrier to primary care use than was cultural/religious beliefs about mental illness and cost of services [24]. Our study team has developed a tool to evaluate competencies of primary care workers trained in mental health [25, 26], which we deployed in mhBeF as a result of these findings.

### Stigma and social distance

Results from this formative research showed that stigma towards PWSMDE is pervasive in the communities of focus. Clear examples ranged from verbal insults and attacks, to exclusion, exclusion by association, physical abuse and violations of human rights. Local idioms of distress and insults are used to refer to PWSMDE. In addition, they are mistreated and their families’ reported experiences of daily embarrassment and shame. PWSMDE reported that they experienced negative treatment ranging from being mocked and teased to being avoided, denied jobs, chained, and beaten. Some participants in the study believed that PWSMDE could never recover from their condition. A law enforcement officer reported that the rights of PWSMDE are being denied. He explained that they are being denied jobs, an assertion that was later confirmed by other respondents.

Additionally, as reported elsewhere, participants in the formative research named health care workers as purveyors of stigma towards PWSMDE [27–29]. This finding serves as a reminder that integration of mental health in primary care may lead to stigma by health care workers. The integration of mental health care into primary healthcare services in South Africa saw many general health care providers being exposed to patients with mental disorders and reported a high level of stigma and discrimination amongst general health care workers [30]. Another finding about stigma of note is that mental health workers are also stigmatized, and referred to as the “Crazy Doctors,” which has been observed in other settings [29].

Based on these findings, stigma and discrimination efforts to introduce mental health services will require attention on how to mitigate stigma so it does not present a barrier to access or represent a burden on persons with mental health conditions and their families. In particular, this study documents stigma towards persons with mental illness by their relatives, their health care providers and the community. Introducing services and enhancing access to care may require a concomitant anti-stigma program that targets the various sources of stigma at the community, individual and facility levels. Efforts to engage service users in these anti-stigma activities holds promise in Liberia, as evidenced by the formation of a user group “Cultivation for Users’ Hope” [31].

### Limitations

The study has limitations which should be taken into account when considering generalization of findings. There were some cadres of health workers (e.g., pharmacist, midwife) with whom we only conducted a KII. This may lead to bias with regards to attributing beliefs to these professions that may be idiosyncratic of the individuals interviewed. Although we picked a region that exemplified a number of common characteristics for rural regions of the country, there may be cultural, economic, and infrastructure differences across regions that would dampen the relevance of findings presented here. In addition, the qualitative data was only collected from Sinoe and therefore may not be applicable to other county health systems. Moreover, the Ebola virus disease outbreak has had differential impacts on health services across the country that would also impact the relevance of our findings.

## Conclusions

This study echoes many of the challenges in the development of primary care mental health programs in LMICs. The results show that developing effective primary care-based mental health services in a post-conflict, West African, low resource setting will require at least three transformations. First, to address the lack of reported self-efficacy to provide mental health care, there is a need for increased knowledge and clinical skills through effective training programs. Second, the poor infrastructure identified through the health facility profiles is a barrier to delivery of mental health services. In particular, lack of any physical space for consultations and therapy may impede diagnosis and care. Third, stigma was a prominent theme and this suggests the need for interventions to improve attitudes toward people with mental illness, as well as attitudes toward their family members and mental health care providers. This requires mental health education at the community and health facility levels as well as ensuring continuous positive engagement with mental health service users.

## Footnotes

1 Health organization refers to district/county level policy makers, planners, service heads, coordinators or administrators who provide support through supervision.

2 Health facility refers to frontline primary health care workers, e.g.: mental health workers, nurses, physician assistants, midwifes, pharmacists and support workers.

3 Frustration in the Liberian English can cover a range of emotions and reactions from disappointed with how life is going to severe depression to giving up on life.

4 African science generally refers to traditional or cultural rituals, witchcraft or casting of spells
